# Supplementary material for: Raw Cow’s Milk Reduces Allergic Symptoms in a Murine Model for Food Allergy—A Potential Role for Epigenetic Modifications
Source: Nutrients. 2019 Jul 25;11(8):1721. doi: 10.3390/nu11081721 (PMC6723026; doi:10.3390/nu11081721)
Supplement: Supplementary file 1 [file nutrients-11-01721-s001.pdf]

Supplementary Materials: The following are available online at www.mdpi.com/xxx/s1, Figure S1: Acetylation patterns of histone H3 were comparable to histone H4 in splenocyte derived CD4+ T cells, Figure S2: No differences between groups observed for histone H3 acetylation in MLN, Table S1: Buffers used for ChIP, Table S2: Primers used for qPCR.

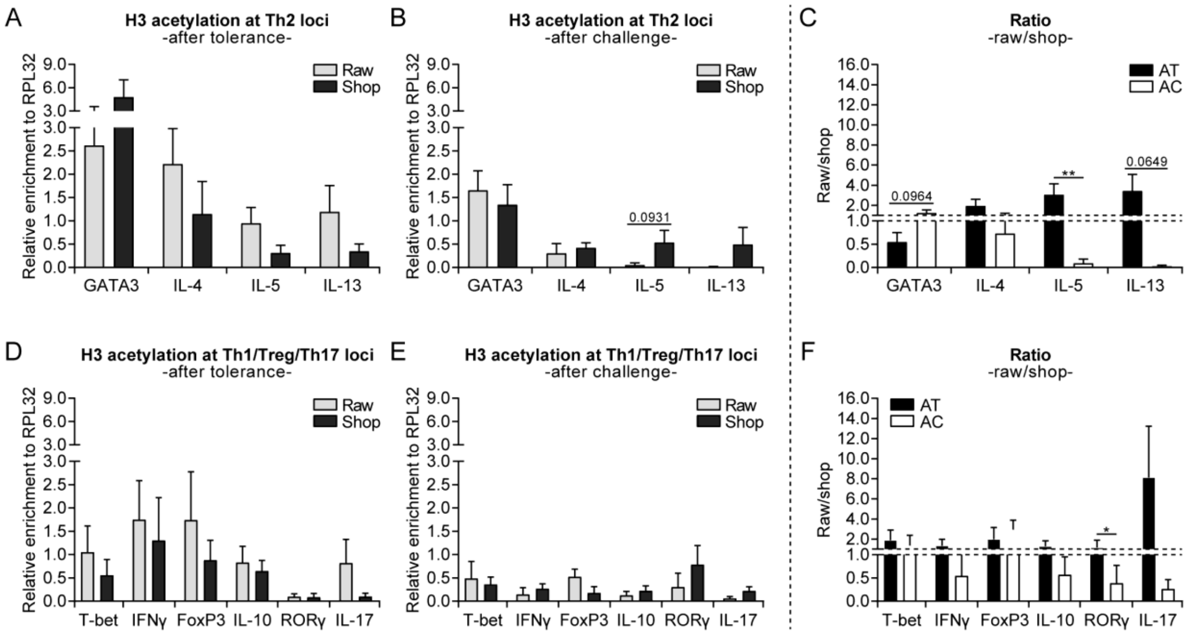

Figure S1. Acetylation patterns of histone H3 were comparable to histone H4 in splenocyte derived CD4+ T cells. (**A**) Histone H3 acetylation at Th2 loci after the tolerance induction period (day -1), (**B**) after both challenges (day 34) and (**C**) the raw milk/shop milk ratio. (**D**) Histone H3 acetylation at Th1/Treg/Th17 loci after the tolerance induction period (day -1), (**E**) after both challenges (day 34) and (**F**) the raw milk/shop milk ratio. Histone H3 acetylation status was determined by means of chromatin immunoprecipitation in CD4+ T cells derived from splenocytes of raw milk and shop milk treated mice. Results are expressed as relative enrichment after normalization to RPL32 as mean ± SEM, $n = 6$/group. $^{*}P < 0.05$, $^{**}P < 0.01$ as analyzed with a Mann-Whitney test. An unpaired two-tailed Student's t-test was used for IL-4, IL-5, IL-13, IL-10 (after tolerance), GATA3 (after model) and GATA3 (ratio raw/shop) since data obtained normality. Raw, raw cow's milk; shop, shop milk; AT, after tolerance; AC, after challenge.

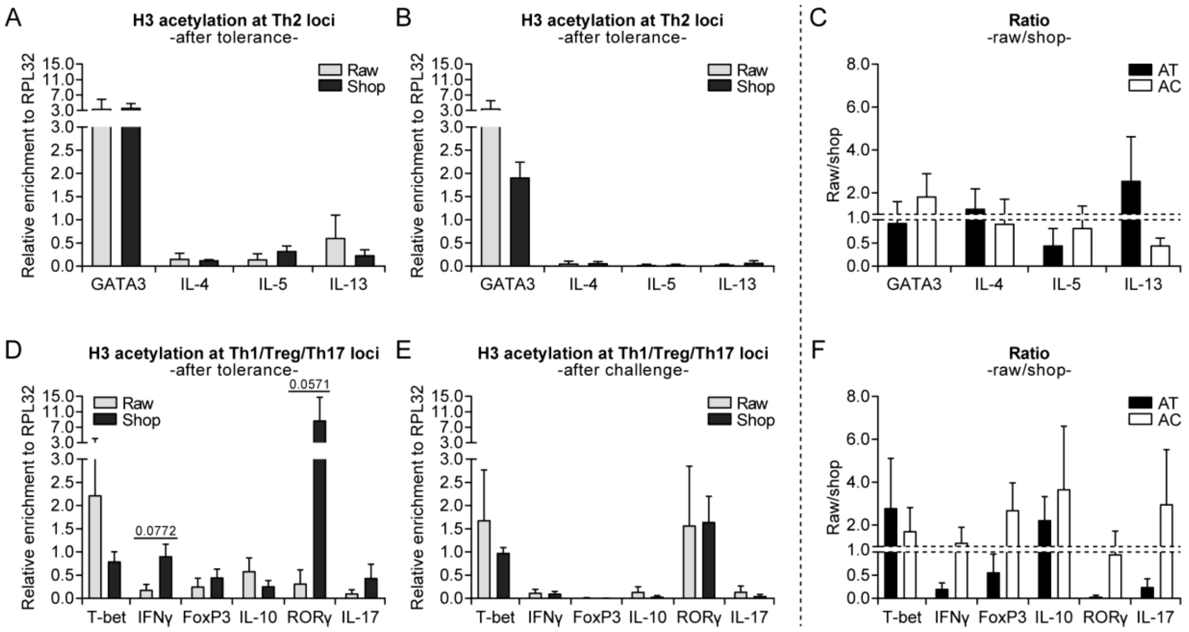

Figure S2. No differences between groups observed for histone H3 acetylation in MLN. (**A**) Histone H3 acetylation at Th2 loci after the tolerance induction period (day -1), (**B**) after both challenges (day 34) and (**C**) the raw milk/shop milk ratio. (**D**) Histone H3 acetylation at Th1/Treg/Th17 loci after the tolerance induction period (day -1), (**E**) after both challenges (day 34) and (**F**) the raw milk/shop milk ratio. Histone H3 acetylation status was determined by means of chromatin immunoprecipitation in MLN of raw milk and shop milk treated mice. Results are expressed as relative enrichment after normalization to RPL32 as mean ± SEM, n = 3-6/group. No significant differences were observed. Raw, raw cow's milk; shop, shop milk; AT, after tolerance; AC, after challenge; MLN, mesenteric lymph nodes.

Table S1. Buffers used for ChIP.

**Lysis buffer I**	For 50 mL
5 mM PIPES pH 8	0.5 mL 0.5 M PIPES pH 8
85 mM KCl	1.4 mL 3 M KCl
0.5% NP40 (Igepal-CA630)	0.25 mL Igepal (100%)
Protease inhibitor cocktail tablets	One tablet

**Lysis buffer II**	For 50 mL
10 mM Tris-HCl pH 7.5	0.5 mL 1 M Tris-HCl pH 7.5
150 mM NaCl	1.5 mL 5 M NaCl
1% NP40 (Igepal-CA630)	0.5 mL Igepal (100%)
1% DOC (Natriumdeoxycholat)	0.5 g
0.1% SDS	0.25 mL 20% SDS
1 mM EDTA	0.1 mL 0.5 M EDTA pH 8
Protease inhibitor cocktail tablets	One tablet

**Wash buffer I**	For 50 mL
20 mM Tris-HCl pH 8	1 mL Tris-HCl pH 8
150 mM NaCl	1.5 mL 5 M NaCl
2 mM EDTA	0.2 mL 0.5 M EDTA pH 8
0.1% SDS	0.25 mL 20% SDS
1% Triton X100	0.5 mL Triton X100

**Wash buffer II**	For 50 mL
20 mM Tris-HCl pH 8	1 mL Tris-HCl pH 8

500 mM NaCl	5 mL 5 M NaCl
2 mM EDTA	0.2 mL 0.5 M EDTA pH 8
0.1% SDS	0.25 mL 20% SDS
1% Triton X100	0.5 mL Triton X100

**Wash buffer III**	For 50 mL
10 mM Tris-HCl pH 8	0.5 mL Tris-HCl pH 8
1% IGEPAL CA630	0.5 mL IGEPAL (100%)
1% DOC (Sodium deoxycholat)	0.5 g
1 mM EDTA	0.1 mL 0.5 M EDTA pH 8
0.25 M LiCl	1.25 mL 10 M LiCl

**1 x TE**	For 50 mL
10 mM Tris-HCl pH 8	0.5 mL Tris-HCl pH 8
1 mM EDTA	0.1 mL 0.5 M EDTA pH 8

**Elution buffer**	For 25 mL
1% SDS	1.25 mL 20% SDS
0.1 M $NaHCO_3$	2.5 mL 1 M $NaHCO_3$

Table S2. Primers used for qPCR.

**Locus**	**Sequence forward**	**Sequence reverse**
RPL32	TCA TTT CTC AGG CAC ATC TT	ACT CAC CGT AAA ACA GAT GG
IL-4	TCT GCC TCC ATC ATC CTT CT	ACA CCA TAA TCG GCC TTT CA
IL-5	ACC CTG AGT TTC AGG ACT CG	TCC CCA AGC AAT TTA TTC TCT C
IL-10	CGA CCA GTT CTT TAG CGC TT	TGT GGC TTT GGT AGT GCA AG
IL-13	CAA CAA AGC AGA GAC CAG GG	CAG AGC CAG TGA GAG AAC CA
IL-17	TGG TTC TGT GCT GAC CTC AT	GCT CTC CCT GGA CTC ATG TT
GATA3	CAC TCG GAT TCC TCT CTC CC	CCA GGA GAG GGG TCG TTT AA
T-bet	CAC TGG TCC ACT GCT CTC TC	GAG ATG TCC GGT GGT GTC TC
RORγ	TGG GGT GCC TGT CAT CAT AC	TGA GAA CTT GGC TCC CTG TC
FoxP3	GAC TCA AGG GGG TCT CA	TTG GGC TTC ATC GGC AA
IFNγ	CAT ACC CTT TCC TTG CTT TTC	TTG TGG GAT TCT CTG AAA GCA

All oligonucleotides were synthesized by Metabion (Planegg, Germany).
